# Multimodal Management of Aggression in Dementia Among Geriatric Patients: A Comprehensive Overview With Particular Emphasis on Pharmacotherapy and Behavioral Interventions

**DOI:** 10.7759/cureus.101807

**Published:** 2026-01-18

**Authors:** Karolina Lichwala, Sara Szukalska, Marta Karczewska, Angelika Samborska, Barbara Balajewicz, Kamil Wróblewski, Lukasz Siwek, Paulina Wróblewska, Karolina Szalata

**Affiliations:** 1 General Medicine, Medical University of Silesia, Katowice, POL; 2 Orthopaedics and Traumatology, Dr. Tytus Chałubiński Specialist Hospital, Radom, POL; 3 General Medicine, Poznan University of Medical Sciences, Poznan, POL; 4 General Medicine, Szpital Miejski w Zabrzu, Zabrze, POL; 5 General Medicine, Jan Mikulicz-Radecki University Clinical Hospital, Wroclaw, POL

**Keywords:** aggressive behavior, dementia treatment, geriatric patient, multimodal treatments, psychomotor agitation

## Abstract

Agitation and aggression are among the most common and clinically challenging behavioral and psychological symptoms of dementia in older adults. These manifestations worsen patients’ daily functioning and increase the likelihood of hospitalization and long-term care placement. In addition, it imposes a significant burden on caregivers and healthcare systems. Their presentation is heterogeneous and reflects the complex interactions among neurodegenerative changes, medical comorbid conditions, environmental stressors, and the quality of care and communication. This review summarizes current approaches to the management of aggression in dementia, with a particular focus on multimodal strategies integrating behavioral, systemic, and pharmacological interventions. Emphasis is placed on non-pharmacological measures, which are widely regarded as first-line treatment due to their favorable safety profile and their potential to achieve sustained clinical benefits. These interventions include individualized behavioral strategies, environmental adaptations, structured daily activities, and caregiver education programs. Pharmacological treatment is discussed as a complementary option, appropriate primarily in cases of severe or persistent aggression or when there is an imminent risk to the patient or others. The review highlights the importance of cautious prescribing and using medications, limiting treatment duration, and regularly reassessing therapeutic effectiveness in relation to adverse effects, particularly in geriatric patients with multimorbidity. We also emphasize the need for an individualized approach, adjusted to the dementia subtype, overall medical condition, and prior treatment response. Ethical considerations are also addressed, including respect for patient dignity, minimization of coercive measures, and the responsibility of care institutions to create environments supportive of persons living with dementia. The available evidence supports a patient-centered, multimodal approach as the most balanced and effective strategy for managing aggression in dementia. Further pragmatic research is obligatory to refine integrated care models and to evaluate their effectiveness across diverse clinical settings.

## Introduction and background

Aggression and agitation in dementia are common and severe neuropsychological symptoms as part of behavioral and psychological symptoms of dementia (BPSD). It includes aggressive behavior, excessive mobility, anxiety, and irritation, placing a significant burden on patients and their caregivers [1]. Dementia is, by definition, a neurodegenerative process in which progressive damage to cortical and limbic structures and disorders of neurotransmitter systems lead to a gradual loss of cognitive function, but also to numerous symptoms [2]. Studies indicate that BPSD affects almost all patients with dementia at various stages of the disease, and symptoms of agitation and aggression may occur regardless of the subtype of dementia, which translates into a higher risk of early placement in a care facility and deterioration in functioning [1].

According to WHO data, approximately 57 million people worldwide were living with dementia in 2021. Furthermore, 10 million new cases are reported annually, making this condition one of the key global public health issues. Although the WHO does not publish separate global statistics on agitation and aggression, clinical data indicate that these symptoms occur in a significant proportion of patients with dementia, affecting about one-third of patients in long-term care and even more than half during the course of the disease [3]. The pathophysiology of aggression in dementia is complex. It involves neuronal degeneration, leading to neurotransmitter imbalances and changes in areas responsible for impulse control, often resulting in inappropriate behaviors [2]. In addition, somatic factors such as pain, infections, metabolic disorders, and sleep disturbances exacerbate BPSD, including aggression, as confirmed by the positive correlation between pain and the severity of agitation in people with dementia [4]. The clinical and social consequences of aggression and agitation in geriatric patients with dementia are enormous. These mentioned behaviors increase the risk of injury to patients and caregivers, impair quality of life, accelerate the need for placement in care facilities, and generate high health and social care costs [1]. Due to the heterogeneous biological, psychosocial, and environmental determinants of aggression in dementia, a multimodal approach combining pharmacological and behavioral methods is considered the most rational and practical in clinical practice. Environmental and psychosocial interventions are proposed as the first-line treatment, and pharmacotherapy is recommended for use on an ad hoc basis, taking complete account of adverse effects and the individual needs of the patient [5]. Current clinical guidelines confirm this model of care: National Institute and Care Excellence (NICE) recommendations indicate that before deciding on pharmacological treatment, a comprehensive assessment of somatic and environmental causes of agitation should be carried out, psychosocial and environmental strategies should be implemented, and antipsychotic drugs should only be used for severe symptoms and risk of harm, at the lowest effective doses and for the shortest possible time [5]. Carefully planned multimodal regimens, combining pharmacotherapy with non-pharmacological treatment methods, emphasize the need to balance benefits and risks and to evaluate effects during therapy continuously [6]. In clinical practice, a sequential approach to the treatment of aggressive behavior is increasingly recommended, starting with non-pharmacological strategies and adding pharmacotherapy only if these prove ineffective, while taking comorbidities and possible interactions into account [7].

Observational data also indicate that in patients who experience both agitation and psychotic symptoms, short-term use of atypical neuroleptics may be justified, provided that there is a plan to gradually withdraw the drugs once the episode has been controlled [8].

Pharmacological treatment of aggression and agitation in patients with dementia is based on limited evidence of efficacy, requires particular caution due to the safety profile and risk of interactions, and its use should be part of a broader clinical approach [7-9].

Methods

This analysis reviews Polish- and English-language scientific literature on dementia accompanied by aggressive behavior in geriatric patients, as well as an overview of therapeutic approaches used in such clinical situations. Particular attention was given to contemporary, evidence-based multimodal management strategies shown to be effective in reducing aggressive behaviors and improving patient functioning.

The literature search was conducted using PubMed, Google Scholar, ScienceDirect, the websites of national and international scientific societies, and current clinical guidelines. Sources published between 2008 and 2025 were included to ensure the review's currency and alignment with contemporary knowledge in geriatrics and dementia care. The review was guided by the following keywords: “dementia”, “aggression”, “agitation”, “geriatric patients”, and “multimodal therapy.” This selection enabled the identification of publications addressing both the epidemiology and characteristics of aggressive behaviors in dementia and the therapeutic strategies available for this geriatric population.

## Review

BPSD are defined as a range of symptoms that often co-occur with dementia. BPSD includes emotional disorders, depression and anxiety, sleep disorders, psychosis, and many behavioral disorders, including aggression and agitation, which are relevant to this study [10]. Patients with dementia very often experience symptoms from the spectrum of behavioral and mental disorders, including psychotic symptoms, agitation, aggression, and other symptoms. One analysis found that approximately 90% of people with dementia experience at least one behavioral symptom during the course of the disease. Psychotic symptoms and behavioral disorders significantly increase the burden on patients and caregivers; they are often associated with a poorer prognosis, faster cognitive decline, an increased need for institutional care, and greater susceptibility to hospitalization. Therefore, aggressive behaviors are a significant health and social problem in the elderly population with dementia [11]. Aggressive behaviors are a common problem among patients with dementia, especially those in home or long-term care settings. The risk of their occurrence increases among patients with severe cognitive impairment, limited communication skills, and reduced mobility. The presence of aggression worsens the patient's well-being and functioning and causes a significant burden on caregivers, increases the risk of errors in care, and negatively affects the patient-caregiver relationship [12,13].

Pathophysiology of aggression in dementia

Dysfunction of cortical and limbic structures that control emotions and inhibit impulsive reactions leads to reduced ability to regulate behavioral responses [11]. Progressive degeneration of cortico-limbic connections and disorders of neurotransmitter systems play a substantial role, intensifying arousal and defensive reactions. In dementias of various etiologies, specific neuropathological patterns further modulate the risk of impulsive behaviors. As a result, the patient gradually loses the ability to control reactions, which contributes to violent episodes of aggression [11].

The most significant role is attributed to the restructuring of the frontal lobe, striatum, thalamus, cerebellum, and corpus callosum. There is a reduction in the volume of gray and white matter in the brain, while neuronal connections in the cerebellum disappear. Additionally, there is decreased efficiency in the synthesis of neurotransmitters, including acetylcholine, serotonin, dopamine, norepinephrine, and gamma-aminobutyric acid [14].

The impact of pain, delirium, and infection on the severity of aggression among patients with dementia

Patients with dementia who also experienced pain showed significantly more neuropsychiatric symptoms. Analyses indicate that patients experiencing pain were 3.8 times more likely to present aggressive behavior than those without pain [15]. In a study of 455 patients with dementia, it was observed that the severity of delirium was linked to a greater number of behavioral symptoms, including aggression [16].

In older people, delirium and dementia often co-occur and can escalate each other, increasing the risk of behavioral disorders [17]. The authors of a meta-analysis analyzed 29 studies involving people aged 65+ years and found that urinary tract infections (UTIs) significantly increase the risk of delirium [18]. Delirium among patients diagnosed with dementia is often associated with behavioral disorders, indicating that UTIs may be a contributing factor to these problems, resulting in an increased risk of impulsivity or aggression [18]. It has been shown that in older people, UTIs often manifest as delirium, which in the context of dementia can exacerbate behavioral disorders, including aggression [19].

Diagnosis of aggression: assessment and monitoring

In the population of people with dementia living in residential facilities, agitation and aggression affect 40%-85% of patients [20]. The traditional Cohen-Mansfield Agitation Inventory (CMAI) questionnaire, completed by caregivers, has limitations due to its subjective nature [20]. In 2019, an observational version, CMAI-O (CMAI-Observational), was developed and validated by an independent observer, thereby improving objectivity [20].

In a validation study of 726 nursing home residents, CMAI-O showed a significant correlation with another scale (Pittsburgh Agitation Scale, PAS), confirming its usefulness [21]. In hospital settings, adapting tools (e.g., a shortened version of the CMAI) enables monitoring changes in the behavior of patients with dementia, especially when agitation or aggression may be symptoms of acute illness, pain, or delirium [22]. Tools such as the Neurobehavioral Rating Scale (NBRS), PAS, and Neuropsychiatric Inventory (NPI) in the Spanish version are among the most commonly used scales for assessing agitation and aggression in dementia and demonstrate the highest sensitivity relative to reference standards. The NPI assesses a wide range of neuropsychiatric symptoms, the NBRS focuses on motor manifestations of hyperactivity, and the PAS measures only the severity of agitated and aggressive behaviors, which determine their different clinical applications. Scales such as CMAI and BEHAVE-AD, despite their frequent use, have limited evidence of diagnostic validity, underscoring the need for further research on their validation for the assessment of BPSD [23].

Non-pharmacological interventions and assumptions of multimodal therapy

An analysis of available studies has shown that non-pharmacological interventions remain the safest and most effective first-line method of reducing aggression in dementia, as they not only reduce the frequency of episodes but also improve the quality of care and well-being of patients without the risk of side effects typical of pharmacotherapy [7,24]. A meta-analysis found that sensory interventions, such as massage, therapeutic touch, and multisensory stimulation, significantly reduce agitation by influencing emotion regulation and reducing physiological tension, especially in patients with limited verbal communication [25]. Animal-assisted therapies showed a marked reduction in aggression, especially in people with more advanced dementia, which is explained by the normalizing effect of zootherapy on behavioral responses [26]. Interventions based on a person-centered approach reduced aggressive behaviors by taking into account the patient's individual needs, emotions, and previous experiences, as well as by modifying communication and the organization of the care environment [27]. Music therapy, although showing mixed results, proved helpful in calming patients by stimulating emotional memory and activating well-preserved brain regions, thereby improving mood regulation and reducing agitation [28]. Staff training covering the analysis of aggression causes, communication techniques, and behavior de-escalation strategies had lasting effects, resulting in a reduction in the frequency and severity of aggression [29]. Multicomponent programs combining elements of sensory, environmental, psychoeducational, and person-centered interventions were most effective in a population of patients with recurrent aggression [30]. Interventions based on the patient's life history were more effective because they strengthened the patient's sense of identity and reduced stress resulting from confusion [27]. Although the effects of non-pharmacological therapies are promising, their implementation requires adequate staff training and organizational changes, without which the effectiveness of interventions is significantly reduced in clinical settings [29].

Multimodal therapy, by definition, involves multidirectional approaches to enhance treatment effectiveness. Pharmacological and non-pharmacological interventions, as elements of integrated care, usually produce better results in alleviating aggression and agitation than monotherapy. The inclusion of non-pharmacological methods allows for the reduction of psychotropic drugs, minimizing the risk of adverse effects. A comprehensive approach, integrating medical, psychosocial, and environmental interventions, is currently the most balanced strategy for treating behavioral disorders in dementia [25].

Environmental interventions

Modifications of the environment, including incorporating natural elements such as therapeutic gardens, can significantly reduce agitation and aggressive behavior in people with dementia, mainly by fostering a greater sense of security [31]. The results of the analyses suggest that environmental interventions are more effective when they include social elements, i.e., when the patient comes into contact with others during the activity [31]. Sensory elements such as natural sounds, plant aromas, or tactile stimulation through textures can promote a sense of calm and security and, as a result, reduce the duration of intense episodes of agitation. However, their effects remain variable between individuals [31]. Some studies indicate that internal adjustments of the space can help regulate circadian rhythms and reduce aggression caused by confusion [32]. Avoiding sensory overstimulation helps reduce aggressive behavior by reducing cognitive overload in patients.

A friendly interior design, with wide corridors, an intuitive room layout, and clear signage, reduces frustration and helps people with dementia avoid the chaos that often leads to aggression [32]. Research on multisensory environmental interventions reports that integrating gardens, aromatherapy, and music reduces agitation and improves quality of life, though these effects depend on the patient's individual needs [33].

A holistic approach to environmental modification can create a stable, predictable environment that reduces the risk of agitation and aggression in patients with dementia's orientation and comfort [31].

Behavioral and Psychosocial Approaches

A review of the literature shows that the Antecedent-Behavior-Consequence (ABC) model is a practical tool for identifying triggers of aggression and designing targeted interventions, as it allows the cause (A) to be separated from the behavior (B) and its consequences (C), which facilitates changes in the environment or in the caregiver's response [34]. Implementation studies show that systematic ABC analysis in patients with dementia allows for the rapid detection of recurring triggers and thus reduces the number of aggressive episodes [34].

Behavioral interventions that use behavior modification techniques reduce the frequency of aggressive behaviors and improve staff cooperation [35]. Person-centered approaches, including validation techniques, help reduce hostility and improve compliance in the patient-caregiver relationship [36]. Reminiscence (life-story work) is used with some patients and has resulted in decreased agitation when conducted regularly and properly [37,38].

Occupational therapy based on the Tailored Activity Program (TAP) model, adapted to the patient's remaining skills and interests, effectively reduces behavioral symptoms, including aggression, mainly when supported by qualified therapists [34,39]. It is worth noting that regular staff training in communication techniques and psychoeducational interventions for caregivers is essential, as it improves the quality of therapy while reducing psychotropic medication doses [35,40].

Sensory-Based Interventions

Music therapy often provides rapid, short-term calming and mood improvement in people with dementia. Still, these effects can vary, depending on the form of treatment and how it is implemented [28,41]. Studies indicate that individually selected music can mainly reduce verbal manifestations of agitation [41,42].

Aromatherapy has shown mixed results: some studies and reviews have demonstrated short-term positive effects on agitation and sleep, but meta-analyses and systematic reviews indicate limited and heterogeneous quality of evidence [43,44]. Single- and multicenter cohort studies suggest that short courses of aromatherapy may reduce agitation severity in some patients. Still, the effects are heterogeneous and depend on the type of oil, dose, route of administration, and characteristics of the study population [43,45]. Massage and tactile interventions show promising results for reducing agitation symptoms, especially when delivered in regular sessions (e.g., head or foot massage) [46,47]. Randomized trials of home massage performed by caregivers show that with proper training, this intervention can reduce both symptoms of severe depression and agitation, while reducing the caregiver's burden [46]. Multisensory approaches often work better than single techniques because they simultaneously address multiple mechanisms underlying aggressive behavior [41,43]. Sensory interventions generally have a favorable risk profile; however, aromatherapy and massage may involve potential contraindications (e.g., skin hypersensitivity and asthma) that must be considered when planning therapy [43,44]. In summary, sensory-based methods are a valuable component of a multimodal treatment plan for aggression in dementia: they are low-invasive, adjustable, and can improve well-being and reduce episodes of agitation, provided that they are individualized, regularly monitored, and supported by appropriate staff training [28,43,46].

Training and Support for Caregivers

Psychoeducational programs that provide caregivers with knowledge about the neuropsychiatric mechanisms of dementia and ways to identify triggers of aggression significantly increase their competence and contribute to reducing problematic behaviors in patients [40]. The intervention's effectiveness is reinforced by training modules on communication techniques, de-escalation, and behavioral analysis, which minimize caregiver stress and limit the risk of aggression escalation [35]. Activity programs tailored to the patient's individual capabilities further reduce caregivers' burden and decrease the frequency of BPSD episodes by improving engagement and a sense of purpose in the activities performed [39].

Biological interventions

Electroconvulsive Therapy

A review of documented studies indicates that electroconvulsive therapy (ECT) can be effective in patients with dementia who exhibit severe aggression or agitation that is resistant to other treatments [48]. In the analyzed reports (case reports, case series, retrospective, one-cohort, and one case-control study), a significant improvement was noted in most patients. The authors report that approximately 88% of patients described experiencing a reduction in agitation/aggression after ECT [48].

The reported side effects were primarily mild or transient, and in many cases, no serious complications were observed, suggesting that ECT is also well tolerated in older people with dementia [48].

Pharmacological treatment of aggression coexisting with dementia 

General Principles for the Use of Psychotropic Drugs in the Geriatric Population

Pharmacotherapy should be considered after implementing and trying non-pharmacological interventions. The choice of drug must be adjusted individually, taking into account the type of dementia, age, comorbidities, concomitant pharmacotherapy, and the risk of adverse effects. The lowest effective dose should be used for the shortest possible time. Regular assessment of benefits and risks, adjusting the dose or discontinuing the drug, is also necessary even if the patient's condition stabilizes. It is essential to monitor the patient for adverse effects and reactions, including changes in behavior, sedation, cardiovascular and motor disorders, and possible cognitive impairment.

Medications Used in the Treatment of Aggression

Antipsychotic drugs: Atypical antipsychotic drugs, such as risperidone or quetiapine, are the most commonly used substances in situations of acute agitation or aggression in patients with dementia, but their effectiveness is moderate and often depends on the clinical context [49]. Many randomized studies have shown that risperidone can reduce the severity of agitation in the short term, but the benefits are small and do not always outweigh the potential risks of side effects (e.g., metabolic syndrome and neurological and cardiac complications) in older people with dementia [7,50].

Meta-analyses indicate that although antipsychotics may bring about a slight improvement in some forms of agitation, their overall effectiveness in reducing aggression in the long term is negligible. Pharmacodynamic and pharmacokinetic interactions are another factor limiting their use [49]. Among atypical antipsychotics, aripiprazole has been studied as potentially milder in terms of adverse effects, but the results do not confirm its superiority in effectiveness over other substances [50]. Short-term use of antipsychotics may be clinically justified in selected cases, provided that side effects are monitored continuously and the need for continued treatment is assessed regularly [7]. The Clinical Antipsychotic Trials of Intervention Effectiveness-Alzheimer’s Disease (CATIE‑AD) study was a multicenter, randomized, double‑blind, controlled trial designed to evaluate the effectiveness and safety of atypical antipsychotic medications in outpatients with Alzheimer’s disease exhibiting psychosis, agitation, or aggressive behaviors [51]. The study demonstrated that these agents provided only modest improvement in agitation and psychotic symptoms, with no single drug demonstrating clear superiority [51]. Furthermore, treatment with atypical antipsychotics was associated with adverse effects on cognitive function and a safety profile that underscores the need for cautious risk-benefit assessment in this vulnerable population [52,53].

Classic neuroleptics show only short-term benefits in reducing agitation and aggression in people with dementia, and evidence of their effectiveness is limited and inconclusive [54]. The use of first-generation drugs (e.g., haloperidol and chlorpromazine) is associated with a significant risk of adverse effects, extrapyramidal and vegetative symptoms, and cardiovascular complications, as highlighted in systematic reviews [55].

Antidepressants: In the dementia population, clinical trials have shown that selective serotonin reuptake inhibitors (SSRIs), especially citalopram, may have a small but statistically significant effect on reducing agitation and aggression compared with placebo, making them a potential alternative to some antipsychotic medications for milder symptoms [56]. The results of the randomized Citalopram for Agitation in Alzheimer's Disease (CitAD) study confirmed that adding citalopram to standard care in people with Alzheimer's disease and agitation can lead to a reduction in symptom severity and a decrease in caregiver distress. However, it was also associated with cognitive impairment and the risk of QTc prolongation, which, especially at higher doses, limits its clinical safety [57]. Reviews show that other antidepressants, including sertraline and trazodone, do not consistently outperform placebo in significantly reducing agitation or aggression in dementia, which means that the choice of drug should be based on the patient's overall profile [56]. SSRIs generally have a more favorable tolerance profile compared with antipsychotics. They are commonly known for having a lower risk of sedation and extrapyramidal symptoms. However, their use may be associated with an increased risk of falls and cardiac problems [58]. A limitation of antidepressants in the context of agitation is that their therapeutic effect is often delayed, meaning that they may be less valuable in violent, intense episodes of aggression [56].

Anticonvulsants and mood stabilizers: Anticonvulsants, such as sodium valproate and carbamazepine, have been studied as mood stabilizers and potential agents for alleviating impulsive behaviors in patients with dementia; however, systematic reviews and clinical data indicate that their effectiveness in reducing aggression is limited and often unpredictable [6]. The mechanisms of action of antiepileptic drugs include modulation of the GABAergic and glutamatergic systems, which may theoretically stabilize arousal; however, in geriatric patients with dementia, greater sensitivity to adverse effects (e.g., sedation, ataxia, and metabolic disorders) limits their clinical use [59]. Mood stabilizers such as lithium have been analyzed in the context of aggressive behavior in dementia. Still, evidence of their effectiveness is insufficient, and the risk of toxicity and numerous drug interactions means that their use in people with dementia is rare. They are often considered a last-resort therapy [60].

Acetylcholinesterase inhibitors (AChEIs): Current reviews indicate that AChEIs, such as donepezil, galantamine, and rivastigmine, do not show the expected reduction in severe symptoms of agitation or aggression, as assessed as part of BPSD, in clinical trials [61]. The available evidence suggests that the effects of cholinesterase inhibitors on aggressive behavior are relatively small or inconclusive, meaning that improvement in BPSD is usually mild. Clinical evidence is inconclusive regarding the direct effects of AChIs on aggression, so further studies specifically designed to assess behavioral symptoms are needed to determine their role in the treatment of BPSD [61]. Nootropic agents: In a randomized, placebo-controlled trial, memantine did not demonstrate significant efficacy in reducing agitation and aggression in patients with moderate-to-severe Alzheimer’s dementia as assessed by the CMAI. However, secondary analyses revealed a beneficial effect of memantine on overall neuropsychiatric symptoms measured with the NPI, as well as on cognitive outcomes. Memantine was administered as add-on therapy, most commonly in combination with AChEIs such as donepezil, which were continued throughout the study period. The treatment was generally well tolerated, with adverse events occurring at rates comparable to placebo. The most commonly observed side effects included dizziness, headache, confusion, and constipation, without evidence of clinically significant safety concerns [62]. In conclusion, memantine may be considered as part of combined therapy, although its effectiveness in the treatment of agitation and aggression per se remains limited.

Benzodiazepines: Current data from randomized trials and systematic reviews indicate that benzodiazepines, such as lorazepam and alprazolam, do not show consistent or clear efficacy in reducing agitation and aggression in dementia. Their effects, if any, are usually short-lived and insufficient for chronic use [63]. At the same time, the literature highlights a high rate of adverse effects in geriatric patients, such as sedation and balance disorders, which significantly limits the safety profile of these drugs in the population with neurocognitive disorders [64]. Furthermore, the observed paradoxical reactions (including increased aggression) indicate the need for extreme caution and close clinical monitoring when using them [64]. Consequently, current guidelines recommend that benzodiazepines be used only on an ad hoc basis, in minimal doses, and in situations of acute agitation when other therapeutic methods have proven ineffective or contraindicated [63,64].

Other substances used to treat aggression associated with dementia: A selective dopamine D₂/D₃ receptor antagonist, tiaprid, remains an off-label drug used to treat agitation and aggression in dementia. Unfortunately, the lack of current large randomized trials significantly limits the ability to assess its efficacy and safety profile clearly [65]. Melatonin shows only moderate improvement in sleep parameters in patients with neurocognitive disorders, which may indirectly benefit cognitive functioning. Still, current data do not confirm a significant effect on aggression reduction [66]. Consequently, both tiaprid and melatonin should be considered adjunctive agents. They should be incorporated into therapy with caution, ideally in combination with other therapeutic strategies, in accordance with current clinical guidelines [65]. Table 1 below summarizes the key evidence on efficacy, safety considerations, and adverse effects of pharmacological therapies used to manage aggression and agitation associated with dementia in geriatric patients.

**Table 1:** Pharmacological treatment of aggression and agitation coexisting with dementia in geriatric patients BPSD, behavioral and psychological symptoms of dementia.

Pharmacological treatment of aggression and agitation coexisting with dementia in geriatric patients			
Drug class	Examples	Efficacy in aggression	Adverse effects/additional information
Atypical antipsychotics	Risperidone, quetiapine, and aripiprazole	Moderate; short-term reduction of agitation [7,49,50]	Metabolic, neurological, and cardiac risks; continuous monitoring and cautious risk-benefit assessment required [7,50,52,53]
Typical antipsychotics	Haloperidol and chlorpromazine	Short-term benefit; limited evidence [54]	High risk of extrapyramidal, autonomic, and cardiac side effects [55]
Antidepressants - selective serotonin reuptake inhibitors	Citalopram, sertraline, and trazodone	Small but statistically significant effect; alternative for mild symptoms [56]	Risk of QTc prolongation, falls, cardiac issues; delayed onset of effect [56-58]
Anticonvulsants/mood stabilizers	Sodium valproate, carbamazepine, and lithium	Limited and unpredictable [6]	Sedation, ataxia, metabolic disorders, toxicity; last-resort therapy [59,60]
Acetylcholinesterase inhibitors	Donepezil, galantamine, and rivastigmine	Minimal or inconclusive impact on aggression [61]	Mild improvement in BPSD; further studies needed [61]
Nootropic agents	Memantine	Limited efficacy, may be considered as part of combined therapy [62]	Dizziness, headache, confusion, and constipation risk [62]
Benzodiazepines	Lorazepam and alprazolam	Short-lived, inconsistent effect [63]	Sedation, balance disorders, and paradoxical agitation; use only acutely and in minimal doses [64]
Other/adjunctive agents	Tiapride	Limited evidence [65]	Use cautiously, ideally combined with other therapeutic strategies [65]
	Melatonin	Moderate sleep improvement, no confirmed effect on aggression [66]	

The table was developed by the authors based on a synthesis of the available evidence.

Overview of current NICE and American Psychiatric Association guidelines

Current NICE guidelines and recommendations based on the American Psychiatric Association (APA) consistently emphasize the need for a structured assessment of the causes of aggressive behavior and the use of psychosocial interventions as the first line of treatment for patients with dementia [5,67]. Pharmacotherapy should be reserved for situations of significant risk, used at the lowest possible dose for the shortest possible time, and regularly reevaluated for efficacy and safety [5,67]. The guidelines also emphasize the need for involving patients and caregivers in therapeutic decisions, with particular attention to the balance of benefits and potential adverse effects of drugs, especially in the context of diagnoses such as Lewy body dementia [5]. Syntheses of international recommendations clearly indicate that a therapeutic hierarchy based on non-pharmacological interventions, individualization of treatment, and continuous risk assessment is key to reducing the unjustified use of psychotropic drugs in BPSD [68].

Alternative and experimental therapies

*Neuromodulation Interventions (Transcranial Magnetic Stimulation and Transcranial Direct Current Stimulation* *)*

Non-invasive neuromodulation techniques, such as repetitive transcranial magnetic stimulation and transcranial direct current stimulation, show promising effects on cognitive function in patients with Alzheimer's disease, but their impact on aggression remains poorly documented and requires further targeted research [69,70]. Combining brain stimulation with cognitive training may enhance neuroplasticity and provide additional benefits. However, trials to date are small and heterogeneous, limiting the ability to make clear recommendations for reducing agitation [71,72]. Mechanical modulation of cortical executive areas may support impulse control, but its effectiveness depends on stimulation parameters and the patient's clinical profile [69].

Medical Cannabinoids

Cannabinoids have anti-inflammatory and monoaminergic modulatory potential, but the quality of currently available clinical evidence is insufficient to make specific therapeutic recommendations [73]. Although small studies suggest the possibility of reducing agitation and anxiety in patients with severe dementia, their use may be associated with a significant risk of adverse effects, such as sedation, imbalance, and arrhythmias, which limits their clinical application [73].

The importance of long-term and systemic care

Institutional care provides greater organizational stability and access to interdisciplinary teams, which promotes more effective management of aggression in dementia than the home environment. In addition, facilities and home care require standardization of diagnostic tools and systemic training to control and manage aggressive behavior [74]. Integrated care models, combining non-pharmacological interventions, pharmacotherapy, and regular clinical monitoring, have the potential to stabilize symptoms and reduce hospitalizations [75]. Research shows that properly designed environmental conditions, such as space modification, social activity enhancement, and tailored sensory stimulation, can effectively reduce aggressive behavior while improving the quality of life of people in long-term care. From a systemic perspective, it is crucial to implement a patient-centered care model and violence prevention strategies. This approach promotes better recognition of patients' unmet needs, which in turn reduces the use of direct coercive measures and dependence on excessive pharmacotherapy [31,74].

Aggression in people with dementia has a multifactorial etiology, which is why a multimodal approach, combining assessment of causes, non-pharmacological interventions, and cautious use of medication, is recommended in most current reviews and guidelines [7]. Meta-analyses and systematic reviews show that personalized programs, massage, animal interaction, and caregiver training reduce the frequency and severity of agitation and aggressive behaviors; however, these effects are variable and depend on the quality of implementation and the specific characteristics of the population [76].

The strengths of the available research include the growing number of randomized controlled trials and network analyses comparing different non-pharmacological interventions, as well as the consolidation of guidelines emphasizing the priority of such methods. Weaknesses include the heterogeneity of interventions, short observation periods, and the low generalizability of results from studies conducted in specific settings (e.g., nursing homes) [76].

Pharmacotherapy remains necessary in selected, difficult, or life-threatening situations, atypical antipsychotics are most commonly used, and SSRIs are also used to some extent, but evidence of long-term efficacy and safety is limited [77]. The main advantage of pharmacotherapy is its relatively rapid action in reducing severe aggression; the main disadvantage is the significant risk of serious adverse events in the elderly (including stroke, pneumonia, fractures, and cardiovascular disorders), as highlighted by extensive observational studies and meta-analyses [51]. In a multimodal therapeutic model, behavioral methods play an undisputed role. Reviews indicate that caregiver training, environmental modifications, a person-centered approach, and individualized care plans can reduce the need for higher medication doses and improve quality of life. Limitations include the intensity of resources required to implement such programs in clinical practice and the lack of standardized protocols or universal treatment regimen [78]. The most significant research gaps requiring further study include: long-term comparisons of multimodal strategies; optimal sequences and doses of medications in frail older adults with multiple comorbidities; mechanisms of action of specific non-pharmacological interventions; and studies of their scalability in home care and resource-limited settings. There is also a lack of research targeting biological subgroups (e.g., different etiologies of dementia) and larger pragmatic trials assessing the costs and effectiveness of interventions [79].

From a clinical practice perspective, it is recommended to record causes and triggers systematically and to implement personalized, non-pharmacological strategies as first-line. Restrictive, temporary medication use and caregivers' training are also essential to achieve the expected therapeutic effect [76].

In addition, it is worth emphasizing the importance of ethical issues: the autonomy of the person with dementia, the legitimacy of the use of coercion or, sometimes, illegitimate coercion, the supply of drugs, and the availability of therapeutic resources. Ethical conduct requires the explicit involvement of caregivers, documentation of indications, time limitations for pharmacological therapies, and the search for less invasive, effective, and humane solutions [7].

There are strong arguments in favor of prioritizing behavioral methods and exercising caution in the use of drugs; however, full confirmation of optimal multimodal models requires further well-designed pragmatic clinical trials covering both therapeutic effects and safety, costs, and ethical aspects [76].

## Conclusions

Multimodal therapeutic management of aggression in patients with dementia is based on integrating behavioral interventions and targeted pharmacotherapy while assessing triggering factors and modifiable clinical conditions. Non-pharmacological interventions remain the first-line strategy due to their favorable safety profile, documented effectiveness in reducing agitation and aggression, and the possibility of long-term maintenance of effects. Pharmacotherapy should only be used in situations of severe aggression or immediate danger, using the lowest effective doses and regularly assessing the therapeutic benefits against the risk of adverse effects. It is crucial to individualize treatment, taking into account the clinical phenotype of dementia, somatic conditions, and the patient's responsiveness to specific therapies. The development of therapeutic methods should focus on optimizing non-pharmacological programs, creating personalized interventions based on behavioral analysis, and searching for pharmacotherapies with a selective mechanism of action and a higher safety profile. In the future, pragmatic studies are needed to validate comprehensive care models and their effectiveness in diverse clinical contexts.
